# Pulmonary sclerosing hemangioma: report of two cases

**DOI:** 10.1186/1477-7819-10-182

**Published:** 2012-09-03

**Authors:** Chuang He, Hongyang Fang, Yun Liu, Xuequan Huang, Wei Zhen, Li Ren

**Affiliations:** 1 Department of Computed Tomography, The 452nd Hospital of PLA, Chengdu 610061, Sichuan Province, China; 2 Department of Interventional Radiology, Southwest Hospital, Third Military Medical University, Chongqing 400038, China

**Keywords:** Pulmonary sclerosing hemangioma, Clinical, Surgical resection, Biopsy, Computed tomography,Pathology

## Abstract

Pulmonary sclerosing hemangioma (PSH) is a rare benign tumor of the lungs. These tumors are composed of cuboidal surface cells and polygonal stromal cells and show four histological manifestations: hemorrhagic, papillary, solid, and sclerotic. PSH predominantly affects asymptomatic middle-aged women. The tumor often occurs at the intralobar site, and less commonly in the bronchus and mediastinum. PSH is easy to be misdiagnosed preoperatively. In this study, we present in detail the treatment procedures followed for two atypical cases of PSH. Case 1 was a 62-year-old woman bearing a tumor for 15 years. The tumor lesion was found to be located in the oblique fissure of the left lung. PSH was confirmed by surgical resection and postoperative pathological diagnosis. There was no sign of recurrence and metastasis 1.5 years after surgery. Case 2 was a 54-year-old woman diagnosed with bilateral multiple nodules by physical examination. This patient was diagnosed with definite PSH through computed tomography-guided percutaneous lung biopsy. Surgical resection was not performed. The patient also showed no sign of enlarged tumor and metastasis after 2 years of follow-up. Although PSH can be cured by surgical resection, the findings in our cases indicate that surgical resection need not be considered the preferred course of treatment. If PSH is diagnosed before surgery, the patients may survive while bearing the tumor.

## Background

Pulmonary sclerosing hemangioma (PSH) is an uncommon benign tumor, which was first described by Liebow and Hubbell in 1956 [1]. Various scholars have suggested that this tumor originates from vascular endothelial, mesothelial, mesenchymal, epithelial, or neuroendocrine cells. Recently, other reports have provided evidence supporting the theory that PSH originates from type II pneumocytes. Although PSH is categorized as a miscellaneous tumor according to the 2004 World Health Organization classification of lung tumors, its etiology and origin remain controversial. PSH can be easily misdiagnosed preoperatively. PSH can be misdiagnosed as a malignant tumor during intraoperative frozen-section assessment and has a misdiagnosis rate of 25 to 56%, which results in unnecessary extensive surgical procedures and malignant biological behaviors, including local lymph-node metastasis and tumor recurrence [2-7]. Definite diagnosis of cases of bilateral multiple PSH is usually possible after postoperative histopathological examination. Cases are seldom diagnosed by performing computed tomography (CT)-guided percutaneous lung biopsy because of the insufficient tissue at the puncture site. There are few reports about the PSH developing in the oblique fissure in lungs, with patients bearing such tumors for more than 15 years. Here we report two cases of PSH and explore the clinical management of PSH.

## Case presentation

### Case 1

**Figure 1:**
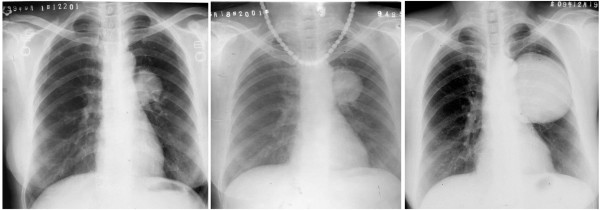
**Chest radiograph of Case 1 obtained from 1999 to 2009.** Chest radiograph of the first patient obtained in 2009 showing a gradual enlargement in the size of the nodule from its size in 1999.

**Figure 2:**
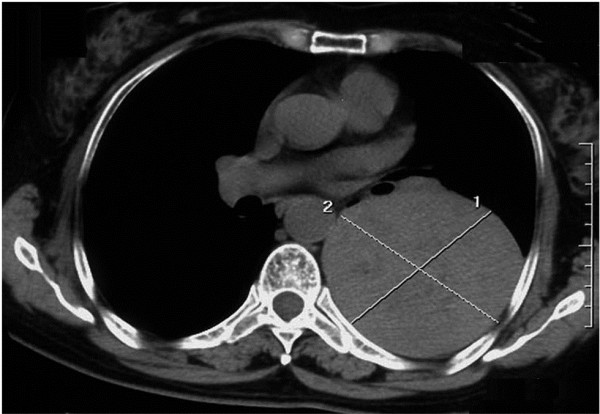
**Computed tomography scan of Case 1 obtained in 2010.** Computed tomography scan obtained in 2010 showing a huge soft-tissue mass with a clear periphery in the left side of the chest, measuring 8.2 cm × 8.6 cm.

**Figure 3:**
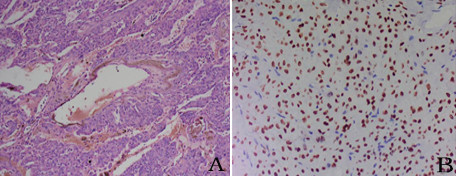
**Pulmonary sclerosing hemangioma was confirmed by surgical resection and postoperative pathological diagnosis. (A)** Two of the typical histological characteristics: hemorrhagic and papillary areas (×100). **(B)** Both the cuboidal surface cells and polygonal cells showed thyroid nuclear factor 1 staining (×200).

**Figure 4:**
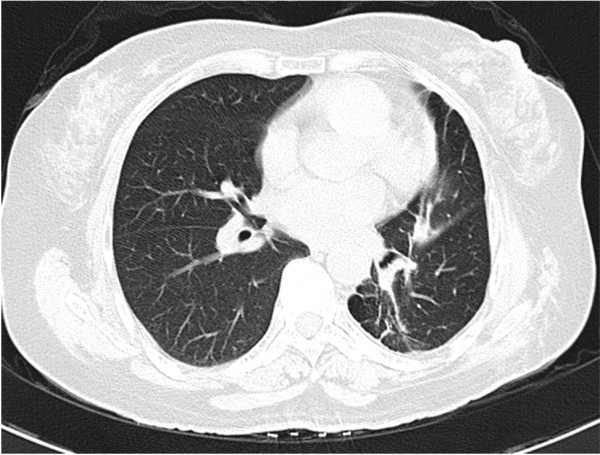
**Computed tomography scan of Case 1 obtained in 2011.** Computed tomography scan obtained in 2011 indicating good expansion of the left lung and a fibrous lesion in the area on which surgery was performed.

The patient was an asymptomatic 62-year-old woman diagnosed with a tumor in the left hilum of the lung in 1995, who had refused to undergo treatment (imaging data of the physical examination had been misplaced). A chest radiograph obtained in 1999 indicated multiple abnormal nodular shadows in the left hilar field; the shadows were approximately 2.5 cm × 3 cm in size, which, in combination with the patient’s description of the original tumor size, indicated that the tumor had not enlarged in sized. A chest radiograph obtained in 2001 showed a slight increase in the lesion size. The tumor measured about 7 cm × 8 cm in 2009 (Figure 1). A CT scan obtained in 2010 showed a giant soft-tissue mass in the left side of her chest, measuring 8.2 cm × 8.6 cm with scattered calcifications at the edge (Figure 2). No positive results were obtained from any of the laboratory examinations conducted when the patient was admitted to the hospital. On intraoperative examination, a well-circumscribed hard tumor measuring 10 cm × 10 cm × 10 cm was found to be located in the fissure of the left lung; the tumor was found to be adhered to the superior lobe, inferior lobe, and posterior chest wall. A vascular endothelial tumor was diagnosed on the basis of intraoperative frozen-section analysis. PSH was confirmed by surgical resection and postoperative pathological diagnosis of a paraffin-embedded section, in which hemorrhagic, papillary areas appeared (Figure 3A). The immunohistochemical results were as follows: thyroid nuclear factor 1 (positive; Figure 3B), cytokeratins (negative), vimentin (positive), CD34 (positive), synaptophysin (negative), and epithelial membrane antigen (positive). The patient was discharged from the hospital after 8 days, without receiving any further adjuvant therapy after the surgery. One and a half years after the surgery, a CT scan indicated good expansion of the left lung and no sign of recurrence and metastasis (Figure 4).

### Case 2

**Figure 5:**
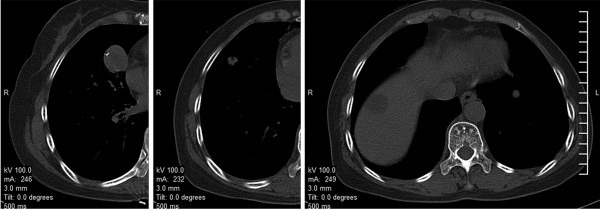
**Computed tomography scan of Case 2.** Multiple pulmonary sclerosing hemangioma in the medial and lateral segments of the middle lobe of the right lung and in the basal segment of the left lower lobe.

**Figure 6:**
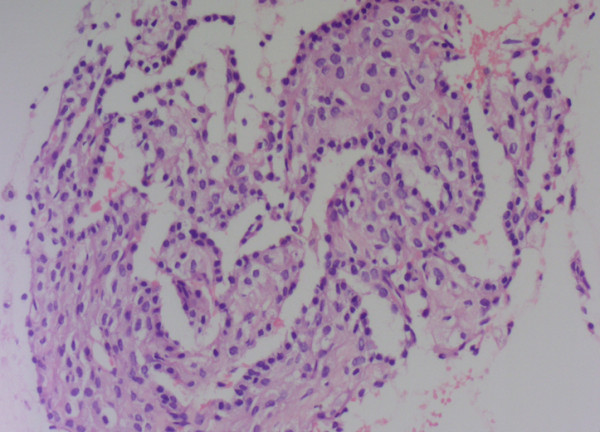
**Pulmonary sclerosing hemangioma was confirmed by CT-guided percutaneous lung biopsy postoperative pathological diagnosis.** Papillary areas: cuboidal cells were on the papillary surface and polygonal cells were in the stroma (×200).

A 54-year-old woman underwent chest CT as a part of physical examination. Round-shaped nodular shadows were observed in the medial and lateral segments of the middle lobe of the right lung and the basal segment of the left lower lobe, with diameters of 2.5 cm, 0.9 cm, and 0.8 cm, respectively. No tumors were observed in the periphery, and point-like calcification was observed in the two nodules in the middle lobe of the right lung (Figure 5). Negative results were obtained in the analysis for all tumor markers. CT-guided percutaneous lung biopsy was performed on the lesion in the middle lobe of the right lung. The biopsy results indicated the development of PSH. Three of the histological characteristics appeared in a paraffin-embedded section (Figure 6). The results of immunohistochemical analyses were as follows: thyroid nuclear factor 1 (positive), synaptophysin (negative), CD56 (negative), CD68 (positive), cytokeratins (positive), and P63 (negative). This patient was therefore diagnosed with bilateral multiple PSH and was discharged from the hospital without any further adjuvant therapy. Two-year follow-up found no increase in the number and size of the lesions.

## Discussion

PSH is a rare benign neoplasm of the lung. The origin of PSH has remained controversial despite extensive studies by many investigators. PSH can occur in any individual in the age group of 4 to 70 years [8], [9]. PSH is predominant in 50 year olds, with a female to male ratio of 5:1 in this patient group [10]. Most of the PSH patients are asymptomatic, and only some show clinical manifestations, including cough, hemoptysis, chest pain, and stuffiness. The observations of the two cases from this study were in accordance with those from previous studies. Both the patients were women with an average age of 50.5 years when they visited a doctor initially, and they were diagnosed with PSH after a physical examination.

PSH is mainly composed of two types of cells: cuboidal surface cells that tend to differentiate into type II pneumocytes, and polygonal stromal cells that have considerable multilineage differentiation potential [11]. In the histopathological analysis, both surface-lining cuboidal cells and pale polygonal cells stained positive for thyroid nuclear factor 1, whereas only some of the cells stained positive for vimentin, epithelial membrane antigen, CD68, and cytokeratins. Wang and colleagues suggested that these differences in morphology and phenotype may be attributed to the differences in the status of the cuboidal and polygonal cells [11]. PSH is histologically characterized by the presence of hemorrhagic, papillary, solid, or sclerotic areas. Most tumors show at least three of these histological characteristics, as also shown in our two cases.

Imaging data for the first case indicated a gradual increase in the lesion size, but the patient did not experience any special discomfort. To date there has been no report of a patient bearing a tumor for 15 years in the oblique fissure area of the lungs. In our first patient, there was no sign of lung and lymph-node metastasis, suggesting that PSH may be showing benign biological characteristics, such as poor growth, non-invasiveness, and nonmetastasis. However, there was evidence indicating that PSH is accompanied by lung or lymph-node metastasis and the etiology of PSH is still unclear, while metastasis of this tumor has no effect on patient survival [4], [5]. Intraoperative analysis indicated a well-circumscribed hard tumor in the oblique fissure of the left lung; the tumor was found to be adhered to the superior lobe, inferior lobe, and the posterior chest wall. Since PSH is known to be composed of cuboidal surface cells and polygonal stromal cells and is derived from type II pneumocytes, we predicted this tumor might have originated in the subpleural lung region of the visceral pleura and then spread to the oblique fissure due to prolonged compression and adhesion to the adjacent structure by the tumor. This case should be differentiated from Castleman disease and pulmonary hamartoma. Castleman disease commonly manifests as an elliptical soft-tissue mass showing a radial or stellate calcification in the central region of the lesion. The presence of fat density within a nodule and the presence of popcorn-like calcifications are specifically indicative of pulmonary hamartoma.

In the second patient, the CT scan showed round-shaped nodular shadows in the middle lobe of the right lung and in the lateral and basal segments of the left lower lobe, with diameters of 2.5 cm, 0.9 cm, and 0.8 cm, respectively. In addition, the periphery was clear and point-like calcification was observed in the two nodules in the middle lobe of the right lung. CT-guided percutaneous lung biopsy was performed only on the nodule in the medial segment of the middle lobe of the right lung. On the basis of the clinical features and the pathological results, this patient was diagnosed with bilateral multiple PSH, which can coexist with lung cancer or can even occur in the same cancerous lesion [12,13]. A similar case has been reported previously [14], but pulmonary lobectomy was performed in that study to diagnose the pathology. The residual nodules showed no changes after 10 years of follow-up. Another study showed tumor recurrence 10 years after resection [3]. Since this condition has multiple probable outcomes, further follow-up should be performed in our patient to thoroughly understand the biological behavior of PSH, which may help determine whether surgical resection or other methods need to be performed. This case should be differentiated from tuberculoma and metastatic tumors. Usually, tuberculoma patients have a history of tuberculosis at a specific location. A metastatic tumor also usually has a history of a primary tumor.

## Conclusions

Surgical resection is currently widely used for treating PSH, and no study has reported distant metastasis and the effect of surgical resection on patient survival. However, PSH predominantly affects middle-aged individuals whose lung function may be bad, and the misdiagnosis rate by intraoperative frozen-section assessment is high, resulting in unnecessary extensive surgical procedures and discomfort for the patients. We therefore suggest that surgical resection should not be considered the preferred strategy for treating single or multiple PSH in the intralobar sites or oblique fissure. If PSH is diagnosed before surgery, and the lesion has no effect on the patient’s respiratory function, the patients can survive bearing the tumor.

## Consent

Written informed consent was obtained from the patients for publication of this Case report and any accompanying images. A copy of the written consent is available for review by the Editor-in-Chief of this journal.

## Abbreviations

CT: computed tomography; PSH: pulmonary sclerosing hemangioma.

## Competing interests

The authors declare that they have no competing interests.

## Authors’ contributions

CH carried out the molecular genetic studies, participated in the sequence alignment and drafted the manuscript. HYF carried out the immunoassays. YL participated in the sequence alignment. XQH and LR participated in the design of the study and performed the statistical analysis. WZ conceived of the study, and participated in its design and coordination and helped to draft the manuscript. All authors read and approved the final manuscript.
